# Toripalimab in Combination With Induction Chemotherapy and Subsequent Chemoradiation as First-Line Treatment in Patients With Advanced/Metastatic Esophageal Carcinoma: Protocol for a Single-Arm, Prospective, Open-Label, Phase II Clinical Trial (TR-EAT)

**DOI:** 10.3389/fonc.2022.878851

**Published:** 2022-04-08

**Authors:** Lei Wu, Yi Wang, Baisen Li, Gang Wan, Long Liang, Tao Li, Jinyi Lang, Qifeng Wang

**Affiliations:** Radiation Oncology Key Laboratory of Sichuan Province, Department of Radiation Oncology, Sichuan Cancer Hospital & Institute, Sichuan Cancer Center, School of Medicine, University of Electronic Science and Technology of China, Chengdu, China

**Keywords:** advanced/metastatic esophageal carcinoma, PD-L1 blockade, concurrent chemoradiotherapy, clinical protocol, toripalimab

## Abstract

Immune checkpoint inhibitor therapy combined with chemotherapy is safe and effective in treating advanced esophageal carcinoma; however, some patients still experience tumor progression and/or metastasis. Whether the addition of radiotherapy to immunotherapy combined with chemotherapy improves the prognosis of patients with advanced/metastatic esophageal carcinoma needs to be investigated. In the present study, we developed a protocol for our clinical trial indicating that toripalimab combined with induction chemotherapy followed by chemoradiotherapy can safely prolong survival in patients with stage IV esophageal carcinoma. This open-label, single-arm, phase II trial will include patients with unresectable stage IV esophageal squamous cell carcinoma who have not received prior systemic therapy. The patients will be treated with two cycles of toripalimab (240 mg, 1 day before chemotherapy, Q3W) combined with induction chemotherapy (paclitaxel, 135–175 mg/m^2^ + carboplatin, area under the curve = 4–6, day 1, intravenous, Q3W). Thereafter, they will undergo two cycles of the aforementioned treatment with concurrent radiotherapy (30–50 Gy in 15–25 fractions), followed by toripalimab (240 mg, day 1, Q3W) for 1 year. The primary outcome measure will be progression-free survival; the secondary outcome measures will include the objective response rate, disease control rate, duration of remission, 1- and 2-year overall survival rates, safety and tolerability, and changes in health-related quality of life. The study protocol was approved by the Ethics Committee of Sichuan Cancer Hospital (SCCHEC-02-2021-021). The trial is underway in accordance with the Declaration of Helsinki.

**Clinical Trial Registration:**

http://www.chictr.org.cn/showproj.aspx?proj=126830, identifier ChiCTR2100046715.

## Introduction

Esophageal carcinoma is a potentially life-threatening malignant disease with a poor prognosis (1). Among the malignant tumors in China, the prevalence of esophageal carcinoma ranks sixth, and its mortality ranks fourth (2). Squamous cell carcinomas form a majority of esophageal cancers in China; most patients with these carcinomas are diagnosed at an advanced stage.

For advanced esophageal cancer, immunotherapy combined with chemotherapy has become the standard treatment recommended by the guidelines. In the phase III trial of pabolizumab or placebo combined with first-line chemotherapy for advanced esophageal cancer (keynote-590) (3), the median overall survival (OS) in the immunotherapy combined with chemotherapy group was more than 12 months, and the efficacy exceeded that of the previous standard first-line chemotherapy. Another phase III clinical study (ESCORT-1ST) showed that carilizumab combined with chemotherapy can significantly prolong the median survival (mOS, 15.3 months vs. 12.0 months) and median progression free survival (mPFS, 6.9 months vs 5.6 months) of patients with advanced esophageal squamous cell carcinoma (ESCC) and has good safety (4).

Despite the combination of immunotherapy and chemotherapy, the prognosis of advanced esophageal cancer is still unsatisfactory. Radiotherapy is a crucial treatment method for patients with advanced esophageal cancer. Theoretically, radiotherapy has a good local tumor control effect, and the improvement of the local control rate is helpful in alleviating symptoms and prolonging survival. Therefore, some studies have tried to add radiotherapy to the first-line treatment of advanced esophageal cancer. Suzuki et al. (5) treated 32 patients with stage IVB esophageal cancer with palliative radiotherapy at an external dose of 30–60 Gy. After treatment, dysphagia in 73% of patients was relieved. Li et al. (6) conducted a retrospective study of 82 patients with heterochronic, oligometastatic esophageal cancer; patients were divided into radiotherapy and non-radiotherapy groups. The median OS of the radiotherapy group (RT) and non-radiotherapy group (NRT) were 14 months and 7 months, respectively. Multivariate analysis showed that treatment mode (RT vs NRT) was an independent prognostic factor for patients with oligometastatic esophageal cancer. In these studies, radiotherapy only was used for local palliative treatment. Whether radiotherapy can achieve a better therapeutic effect in combination with immunotherapy and chemotherapy is an urgent research topic (7–9) However, there are no reports on the combination of radiation with chemoimmotherapy in advanced esophageal cancer.

Although radiotherapy combined with chemotherapy may have benefits, its mechanism of action raises safety concerns because radiotherapy- and immunotherapy-related toxic and side effects might overlap. In addition, the optimal target range, dose fraction schemes, total radiation dose, and timing of incorporation of radiation into the treatment region are unknown. To address this question, we will conduct a single-arm phase II study involving 30 patients with unresectable advanced ESCC. Patients will initially receive a combination of programmed cell death protein 1 (PD-1) inhibitor (toripalimab) therapy and induction chemotherapy, followed by immunotherapy and concurrent chemoradiotherapy (cCRT); eventually, they will be treated with toripalimab as maintenance therapy. Through this trial, we aim to provide preliminary evidence regarding the feasibility of this combination regimen as a first-line treatment option for patients with advanced ESCC.

The main objective of this study is to evaluate the efficacy and safety of triple therapy involving toripalimab in combination with induction chemotherapy followed by chemoradiation in patients with primary stage IV ESCC.

## Methods

### Study Design

This is an open-label, single-arm, single-center phase II clinical trial. Patients with locally advanced or distant metastatic ESCC who have not received prior systemic therapy, including those with primary stage IV ESCC with multiple lymph node metastases (N3) and distant oligometastases (M1; the American Joint Committee on Cancer, 8th edition), will be enrolled (10).

### Patient and Public Involvement

Patients and the general public were not involved in the design, recruitment, and implementation of the study. We have no plans of informing the results of this study to the included patients. However, the results will be disseminated to the applicants in the form of a published article as requested.

### Key Eligibility Criteria

Eligible patients should present histologically confirmed, untreated ESCC considered unresectable (locally advanced or metastatic). In addition, these patients are required to have an Eastern Cooperative Oncology Group performance status (ECOG PS) of 0 to 1, normal organ function, no history of active autoimmune disease, and no history of immune checkpoint inhibitor (ICI) or chemotherapy treatment. Key inclusion and exclusion criteria are listed in **Table 1**.

**Table 1:** Key eligibility criteria for this trial.

Inclusion criteria	Exclusion criteria
Age from 18 to 75 years	Active or untreated CNS metastases, as determined using CT or MRI during screening and prior radiographic assessments
Pathologically confirmed unresectable esophageal squamous cell carcinoma	The site or number of tumor metastases has exceeded the range for oligometastasis
Multiple lymph node metastases (N3) and/or distant oligometastasis (M1)^†^	Uncontrolled cancer-related pain
At least 1 measurable lesion according to RECIST v1.1	Uncontrolled pleural effusion, pericardial effusion, or ascites requiring recurrent drainage procedures (once monthly or more frequently)
ECOG PS 0–1	Uncontrolled or symptomatic hypercalcemia
Life expectancy ≥ 6 months	Bone metastases of the multi-segmental vertebral body, ilium, and other sites
Suited for concurrent radiochemotherapy	Patients with the tendency to exhibit a complete obstruction under endoscopy that requires interventional therapy or surgery for relief of the obstruction
Suited for toripalimab treatment	Stent implanted in the esophagus or trachea
No autoimmune disease	High risk of hemorrhage or perforations due to tumor invasion in adjacent organs (aorta or trachea), or the presence of a fistula
No previous anti-cancer systematic treatment	Severe malnutrition (PG-SGA ≥ 9)
Adequate organ function in accordance with the following:	Allergy to any component of toripalimab, paclitaxel/nab -paclitaxel, or carboplatin
absolute neutrophil count ≥ 1.5 × 10^9^/L	History of or comorbid bleeding disease
hemoglobin ≥ 9 g/dL	Pregnancy or lactation (women)
platelet count ≥ 100 × 10^9^/L	Severe insufficiency of heart, lungs, liver, or kidneys
serum albumin ≥ 2.8 g/dL	Disease of the hematopoietic or immune system, or cachexia
white blood cell count ≥ 4.0 × 10^9^/L	Participation in another interventional clinical study at the same time
total bilirubin ≤ 1.5 ULN; ALT, AST ≤ 1.5 UILN	
serum creatinine ≤ 1.5 ULN	
endogenous creatinine clearance rate ≥ 50 mL/min (Cockcroft–Gault formula)	
blood urea nitrogen within the normal range	
normal thyroid function	

^†^Oligometastasis was defined as ≤5 metastatic lesions in ≤3 metastatic organs. Abbreviations: CNS, central nervous system; CT, computed tomography; MRI, magnetic resonance imaging; RECIST, Response Evaluation Criteria in Solid Tumours; ECOG PS, Eastern Cooperative Oncology Group performance status; PG-SGA, Patient-Generated Subjective Global Assessment; ULN, upper limit of normal; UILN, upper international limit of normal; ALT, alanine transaminase; AST, aspartate transaminase.

### Withdrawal Criteria

Patients will be withdrawn from this study for the following reasons: (1) patients with disease progression, according to Response Evaluation Criteria In Solid Tumors (RECIST) 1.1; (2) patients who experience any unacceptable treatment-related adverse events and cannot continue the study after being evaluated by the study physician; (3) patients that may significantly affect the evaluation of clinical status, for example if they are non-compliant with the research plan, received other treatment, etc.; (4) patients with diseases requiring interruption of treprizumab treatment, such as an allergy, sudden onset of other diseases, and accidents and injuries not related to the disease; and (5) patients exercising their right to withdraw from the trial at any time and for any reason.

### Screening

Patients will be screened within 2 weeks prior to treatment commencement to assess their tolerance to the treatment. Comprehensive information on potentially eligible patients will be collected and recorded during this period. The screening process will include obtaining written informed consent, collection of demographic information and medical history, physical examination, evaluation of ECOG PS score and vital signs, clinical testing (chemistry, hematology, and coagulation), examination of liver and kidney function, and cardiac analyses. Tumor information will be obtained *via* imaging evaluation [computed tomography (CT) or magnetic resonance imaging (MRI)], fibroscopy, esophagoscopy, or positron emission tomography/CT. Eventually, the inclusion and exclusion criteria will be reviewed to make a final judgment regarding each patient’s eligibility.

### Interventions

**Figure 1:**
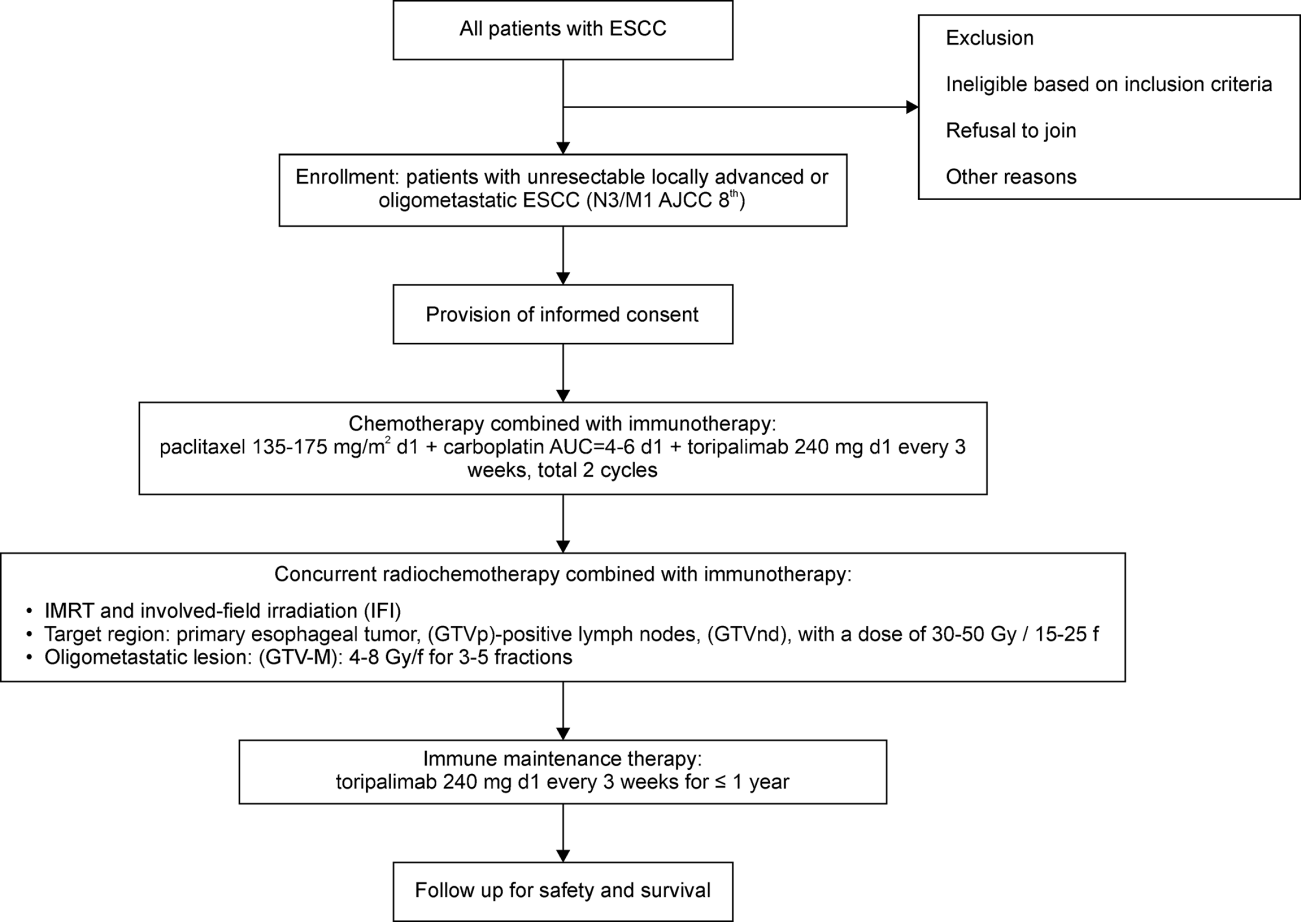
Flowchart of the phase II study. ESCC, esophageal squamous cell carcinoma; AJCC, American Joint Committee on Cancer; d1, day 1; AUC, area under the curve, IMRT, intensity-modulated radiation therapy; GTV-M, gross tumor volume of the primary metastatic lesions; GTV_nd_, gross tumor volume of lymph nodes; GTV_p_, primary gross tumor volume.

Eligible patients will receive two cycles of immunotherapy [toripalimab (240 mg), intravenously infused on the day before chemotherapy] in combination with standard chemotherapy [paclitaxel (135–175 mg/m^2^), day 1 (d1); intravenously infused + carboplatin, area under the curve (AUC) = 4–6, day 1, intravenously infused] every 3–4 weeks. Thereafter, the patients will undergo two cycles of the aforementioned treatment, with concurrent radiotherapy *via* an involved-field irradiation (IFI) technique, thereby targeting only the primary esophageal foci and metastatic lymph nodes, rather than attempting regional prophylactic irradiation of the lymph node drainage area. Stereotactic body radiation therapy (SBRT) may be preferred for some patients with oligometastatic lesions in the bones, lungs, and liver. Eventually, toripalimab monotherapy (240 mg, intravenously infused) will be administered as the maintenance treatment every 3 weeks after the completion of radiotherapy for a maximum period of 1 year. Moreover, the patients will sign an informed consent form to indicate that they understand the purpose and method of the study and will voluntarily cooperate with the trial process; furthermore, they will follow the treatment regimen until progressive disease (PD) or intolerable adverse events (AEs) occur. If AEs of grade 3 occur, the treatment will be suspended, and the AEs will be aggressively managed until the patient’s condition returns to normal or until the AEs have been reduced to grade 1 or 2. The chemotherapy dose may be reduced in the second treatment cycle at the discretion of the investigator. In addition, the medical safety team will review all the safety information during this clinical study. The flow chart of the study is illustrated in **Figure 1**.

Patients will be allowed to withdraw from the study owing to PD or intolerable toxicity as well as upon patient or investigator request. Toripalimab injections may be continued if, in the judgment of the investigator, the treatment remains clinically beneficial for patients with imaging-confirmed PD according to RECIST 1.1. Patients who complete the study treatment will be followed up for survival.

### Follow-up

Patients will undergo tumor assessments at baseline (screening period) and every 6 weeks ( ± 7 days) for the first 12 months after treatment initiation. Patients will be followed up once every 3 months in the first two years, once every 6 months in the third to fifth year, and once a year thereafter. The electronic case report form will be used for data collection and data management. Follow-up examinations will include contrast-enhanced CT scans of the neck, chest, and abdomen, esophagoscopy, abdominal color Doppler ultrasonography, and laboratory analysis of tumor markers in the blood. **Table 2** provides details of the schedule of enrollment, interventions, and assessments.

**Table 2:** Flowchart of enrollment, interventions, and assessments.

Study Phase Therapy	Screening	Treatment visit	Treatment visit	Treatment visit	Treatment visit	Treatment Visit	Treatment Visit	Follow up visit
		I+C cycle 1	I+C cycle 2	I+C+R cycle 3	I+C+R cycle 4	End of RT	Immunotherapy	Follow up
Timepoint	-d14–d0	d1 (w1d1)	d22 (w4d1)	d43 (w7d1)	d85 (w10d1)	w11–w12	w13–w52	
Procedures Visit No	1	2	3	4	5	6	7	8
Informed Consent	X							
Inclusion/Exclusion Criteria	X							
Medical History	X							
Prior cancer therapy	X							
ECOG PS	X	X	X	X	X	X	X	X
Physical Examination	X	X	X	X	X	X	X	X
Vital Signs	X	X	X	X	X			
Weight	X	X	X	X	X	X	X	X
Symptom/Adverse Event Assessment	X	X	X	X	X	X	X	X
Concomitant Medication	X	X	X	X	X	X	X	X
Laboratory tests	X	X	X	X	X	X	X	X
Esophagoscopy	X						X	X
Fibroscopy	X							
Imaging (CT/MRI)	X			X		X	X	X
ECT	X							
Pulmonary function	X							
ECG	X	X	X	X	X	X	X	X
Doppler echocardiography	X			X		X	X	
Histology assessment	X							
QoL-Questionnaires	X			X		X	X	
Exploratory biomarker blood draw	X			X		X		
Survival status							X	X
Review of subsequent therapy							X	X

CT, computed tomography; MRI, magnetic resonance imaging; ECOG PS, Eastern Cooperative Oncology Group performance status; I, immunotherapy; C, chemotherapy; R/RT, radiotherapy; ECG, electrocardiograph; ECT, emission computed tomography; QoL-Questionnaires, quality of life-questionnaires.

### Outcome Measures

The primary outcome of this study is progression-free survival (PFS) among the patients, whereas the secondary outcomes include the objective response rate (ORR), disease control rate (DCR), duration of response (DOR), 1- and 2-year OS rates, patient-reported health-related quality of life, and safety and tolerability of the treatment.

Moreover, we will investigate the potential predictive and prognostic biomarkers, including programmed death-ligand 1 (PD-L1) expression in archived and/or fresh tumor tissue and blood samples obtained before and/or after the completion of the study treatment and/or at the time of PD *via* next-generation sequencing and multicolor immunohistochemical assays. Thereafter, we will assess the relationships between biomarkers, including PD-L1, circulating tumor DNA (ctDNA), and cytokines as well as the therapeutic effect of combination treatment. Furthermore, we aim to investigate the immune microenvironment, immune-related gene expression, and immune-related factors, as well as their associations with disease status and treatment response.

### Safety Assessment

Safety will be assessed based on the observation and documentation of AEs and serious AEs of any grade (according to NCI-CTCAE 5.0) during treatment, laboratory analyses, electrocardiography, physical examination, and ECOG PS scores. Investigators will be responsible for the appropriate measurement of AEs and the determination of causal relationships between AEs and the study drugs.

### Statistical Analysis

All patients who receive the experimental drugs at least once and have had at least one safety evaluation will be included in the Safety Set (SS) analysis. According to the principle of intention-to-treat (ITT) analysis, the full analysis set (FAS) will include data from the last observation of all the cases that had used drugs at least once and were followed up at least once; the entire treatment process cannot be observed until the final results. The FAS data set will be used for fall-out analysis, equilibrium analysis of basic indicators, analysis of the main efficacy indicators, and analysis of safety indicators. The per protocol set (PPS) analysis is a statistical analysis of case data that can meet all the prescribed requirements in accordance with the protocol. This analysis method does not include cases that violate the trial protocol, such as cases lost on follow-up or where patients used prohibited drugs. In this study, the SPSS22.0 software will be used for statistical analysis. Quantitative data satisfies the requirements of normal distribution using the mean ± standard deviation and meets the requirements expressed by median (P25, P75). Qualitative data will be expressed as percentage (%), and a confidence level of 95% will be used for confidential intervals. The Kaplan–Meier method will be used to estimate survival rates and median survival time and to draw the survival curve. The Log-Rank test will be used to compare the survival rate. A Cox regression model will be established to estimate the hazard ratio (HR) between different parting spaces. A two-sided test will be conducted for all statistical tests, and P<0.05 will indicate that the differences were significant.

### Sample Size Calculation

Based on the literature, the mPFS of pabolizumab combined with cisplatin and 5-fluorouracil as the first-line treatment of unresectable locally advanced or metastatic esophageal cancer was 6.3 months (3). Our preliminary work showed that the mPFS of toripalimab in combination with induction chemotherapy and subsequent chemoradiation in the treatment of primary stage IV ESCC was 12.0 months or more. We hypothesized that the mPFS of our trial can reach 12 months. The type I error rate is 5%, and the power is 80%. The sample size calculated by PASS 22.0 was 25 cases (or a minimum of 25 patients). In consideration of a 20% drop-out rate, the final sample size is set at 30 cases.

### Data Collection, Management, and Monitoring

The final patient data should be collected according to the study protocol, including electronic Case Report Form (eCRF) and external data (such as laboratory data), which will be saved by the study sponsor at the end of the study. In the collection of data in eCRF, the instructions in the filling guidance of eCRF should be followed. The researcher is ultimately responsible for collecting and reporting all clinical data entered into eCRF. To ensure accurate data collection, related standard procedures should be followed. The outlier, logic, inconsistency, and integrity of data will be examined. During the study, the supervisor will visit the study center, examine the protocol compliance, define the consistency between eCRF and medical records of the patient, and ensure that the study is done according to related supervision requirements.

## Discussion

To the best of our knowledge, the present clinical trial will be the first to examine the efficacy and safety of triple therapy involving toripalimab in combination with induction chemotherapy followed by chemoradiation in patients with primary stage IV ESCC. Recently, chemotherapy combined with immunotherapy was used as a first-line treatment for advanced esophageal cancer; however, the prognosis of advanced esophageal cancer is still poor. Therefore, more effective treatment options are needed to improve patient survival and prognosis.

Radiotherapy is an important nonsurgical option for treating esophageal cancer; cCRT has become the standard of care for treating locally advanced ESCC (11). However, for treating advanced esophageal cancer, radiotherapy is regarded as a means of palliative treatment. Advanced esophageal cancer is a broad concept, which includes patients with multiple organ metastasis and oligometastatic patients with a limited number of metastatic lesions and fewer metastatic organs. The latter is often considered to have a better prognosis. Therefore, the treatment of oligometastatic esophageal carcinoma differs from the palliative treatment of advanced esophageal cancer; the former includes more aggressive treatment modalities. For instance, radical radiotherapy with a bioequivalent dose of 10–60 Gy revealed remarkable benefit in the survival of patients with heterochronic oligometastatic esophageal cancer (6). Recent evidence suggests that ICIs act synergistically with either chemotherapy or cCRT to exert antitumor effects. Both chemotherapy and radiotherapy can upregulate PD-L1 expression by releasing cytokines and other inflammatory molecules (12, 13) and sensitizing tumors to PD-1/PD-L1-mediated therapy. In this context, chemotherapy and radiotherapy serve as synergistic therapies for immunotherapy. The removal of cancer cells *via* chemotherapy and/or radiotherapy can cause antigen release, thereby converting a less immunogenic or immunosuppressed tumor into an immunogenic tumor (14). In addition, radiotherapy can mobilize both innate and adaptive immune responses, induce tumor-specific T-cells, and establish a tumor-specific immune memory, which collectively enhances the effect of radiotherapy, improves local control, reduces metastatic spread, and prolongs OS (15).

Our previous retrospective studies (Not yet published) suggest that the treatment strategy of this study has a good therapeutic effect on patients with oligometastatic esophageal cancer. The possible advantage of this strategy is that induction therapy reduces tumor volume and tumor load by first alleviating symptoms of dysphagia and consequently improving nutrition and general physical health. Furthermore, tumor shrinkage creates favorable conditions for subsequent cCRT, which include considerably reduced target volumes for radiotherapy, alleviation of toxic side effects caused by radiotherapy, lower risk of bleeding and perforation in tumors and the gastrointestinal tract, and better protection of normal surrounding tissue. However, no standard strategies exist for synergistically combining radiotherapy with immunotherapy and chemotherapy in the treatment of advanced ESCC. Further clinical studies are required to explore the optimal target area range, segmentation mode, total dose, and time of administering radiotherapy.

There are several limitations to our study. First, the number of patients enrolled is small. Secondly, this is a single arm and single center study. We expect to have a randomized controlled study that compares the differences between the triple therapy modality with radiotherapy and the immunochemotherapy modality without radiotherapy in the future.

In summary, this clinical trial will attempt to evaluate the efficacy and safety of toripalimab in combination with induction chemotherapy and subsequent chemoradiotherapy as first-line treatment for patients with primary stage IV ESCC. We expect that the results of this phase II study will provide preliminary evidence for further evaluation of combination treatment options for patients with primary stage IV ESCC.

## Data Availability Statement

The original contributions presented in the study are included in the article/supplementary material. Further inquiries can be directed to the corresponding authors.

## Ethics Statement

The studies involving human participants were reviewed and approved by Ethics Committee of Sichuan Cancer Hospital (SCCHEC-02-2021-021). The patients/participants provided their written informed consent to participate in this study.

## Author Contributions

JL designed the investigation and contributed to writing the paper. TL and QW participated in the administration of this study and contributed to writing the paper. JL and GW were involved in obtaining ethical approval. BL, YW, and GW provided essential assistance and gave suggestions in writing this manuscript. LW, BL, and YW performed the research and supervised the study. All authors read and approved the final manuscript.

## Funding

This study was supported by grants from the Sichuan Science and Technology Department Key Research and Development Project Fund [2018SZ0210,2019YFS0378] and the Applied Basic Research Programs of the Science and Technology Department of Sichuan Province [2020YJ0446].

## Conflict of Interest

The authors declare that the research was conducted in the absence of any commercial or financial relationships that could be construed as a potential conflict of interest. However, one drug, toripalimab, was provided for free by Shanghai Junshi Biosciences Co., Ltd., who had no direct role in the design of this protocol or in the collection, analysis, or interpretation of data.

## Publisher’s Note

All claims expressed in this article are solely those of the authors and do not necessarily represent those of their affiliated organizations, or those of the publisher, the editors and the reviewers. Any product that may be evaluated in this article, or claim that may be made by its manufacturer, is not guaranteed or endorsed by the publisher.
